# Corticotropin‐releasing hormone improves survival in pneumococcal pneumonia by reducing pulmonary inflammation

**DOI:** 10.14814/phy2.13000

**Published:** 2017-01-05

**Authors:** Brittney Burnley, Harlan P. Jones

**Affiliations:** ^1^ Institute of Molecular MedicineUNT Health Science CenterFort WorthTexas

**Keywords:** Corticotropin‐releasing hormone, glucocorticoids, inflammation, neuropeptides, *Streptococcus pneumoniae*

## Abstract

The use of glucocorticoids to reduce inflammatory responses is largely based on the knowledge of the physiological action of the endogenous glucocorticoid, cortisol. Corticotropin‐releasing hormone (CRH) is a neuropeptide released from the hypothalamic–pituitary–adrenal axis of the central nervous system. This hormone serves as an important mediator of adaptive physiological responses to stress. In addition to its role in inducing downstream cortisol release that in turn regulates immune suppression, CRH has also been found to mediate inflammatory responses in peripheral tissues. *Streptococcus pneumoniae* is a microorganism commonly present among the commensal microflora along the upper respiratory tract. Transmission of disease stems from the resident asymptomatic pneumococcus along the nasal passages. Glucocorticoids are central mediators of immune suppression and are the primary adjuvant pharmacological treatment used to reduce inflammatory responses in patients with severe bacterial pneumonia. However, controversy exists in the effectiveness of glucocorticoid treatment in reducing mortality rates during *S. pneumoniae* infection. In this study, we compared the effect of the currently utilized pharmacologic glucocorticoid dexamethasone with CRH. Our results demonstrated that intranasal administration of CRH increases survival associated with a decrease in inflammatory cellular immune responses compared to dexamethasone independent of neutrophils. Thus, providing evidence of its use in the management of immune and inflammatory responses brought on by severe pneumococcal infection that could reduce mortality risks.

## Introduction

The use of glucocorticoids to reduce inflammatory responses is largely based on the knowledge of the physiological action of the endogenous glucocorticoid, cortisol. Glucocorticoids can exert their immunosuppressive properties on both cells and its cellular environment (e.g., proteins, membranes, and organelles) (Culpitt et al. 1999). In response to infection, cortisol secretion from the adrenal glands acts in part as a global suppressor of immune and inflammatory responses. This mechanism protects against extended tissue damage and restores homeostatic conditions. However, its use can also result in an increased risk to secondary infections caused by a nonspecific immune suppression.

Cortisol is a human glucocorticoid whose release is mediated by corticotropin‐releasing hormone (CRH). CRH is a neuropeptide released from the hypothalamic–pituitary–adrenal (HPA) axis of the central nervous system. This hormone serves as an important mediator of adaptive physiological responses to stress (e.g., physical, psychological, and environmental). In addition to its role in inducing downstream cortisol release that in turn regulates immune suppression, CRH has also been found at peripheral sites of inflammation (Saunders et al. 2002; Ganceviciene et al. 2009; Rassouli et al. 2011). Previous findings suggest that CRH, through its ligation to its receptors, can modulate cellular immune and inflammatory responses (Sternberg et al. 1992; De Miguel et al. 2011; Sasayama et al. 2011; Wang et al. 2012). However, its mechanisms of action on cellular immune function remain unclear. Importantly, few studies have demonstrated how CRH may impact innate pulmonary immune defenses against bacterial infections (e.g., *Streptococcus pneumoniae*) (Murray et al. 2001; Gonzales et al. 2008; Kim et al. 2011). Determining the mechanism of action of CRH on particular inflammatory cells will provide a novel understanding of mechanisms mediating cellular immune inflammatory responses caused by severe bacterial infections.

*Streptococcus pneumoniae* is a microorganism commonly present among the commensal microflora along the upper respiratory tract. Among the 92 known serotypes of *S. pneumoniae*, few are considered pathogenic. Rather, transmission of disease stems from the resident asymptomatic pneumococcus along the nasal passages. Still a majority of deaths are due to complications from respiratory pneumonia caused by *S. pneumoniae* (De Pascale et al. 2011; Rodgers and Klugman 2011; Jinno and Jacobs 2012). For many years, routine vaccination, antibiotic use, and adjunctive therapies have proven efficacious in certain settings and for certain groups. For example, immunization programs have proven successful in reducing pneumonia and are predicted to reduce child mortality by 30% by 2015 globally (Boyce et al. 1995; Frei et al. 2011). However, the emergence of virulent serotypes not covered by current vaccines and the notable rise in antibiotic resistance raises concern for increased mortality risk caused by *S. pneumoniae*.

Individuals particularly at risk include the very young (<6 months to 3 years), the elderly (>65 years), and the individuals with chronic disease (e.g., emphysema, chronic obstructive pulmonary disease, immunodeficiency) (Sternberg and Licinio 1995; Almagro et al. 2012). The risk associated with these groups is believed to be in part due to their lack of immune competency. During the early stages of *S. pneumoniae* infection, robust innate cellular immune and inflammatory responses play a critical role in eradicating *S. pneumoniae* from the lower respiratory tract as the host's response in preventing persistent infection and disseminating systemic disease. However, under conditions whereby the host immune response fails to clear the ensuing infection, immune‐mediated inflammatory responses can have detrimental effects, particularly where antibiotic treatment is also ineffective. To avoid such outcomes, suppression of inflammatory responses through glucocorticoids is used as an adjunctive therapy during severe infection (Refojo and Holsboer 2009; Wunderink 2009). Dexamethasone is a common adjuvant therapy used to reduce inflammatory responses in patients with bacterial pneumonia (Jain et al. 1991; Jessop et al. 2001; Adcock et al. 2012; Vlahos et al. 2012). The purpose of suppressing inflammatory responses during severe infection is to avoid tissue damage, leading to both sepsis and death. *Streptococcus pneumoniae*‐associated sepsis occurs by dissemination from the initial site of infection (e.g., the lung) due to tissue injury caused by severe inflammatory reactions leading to bacteremia in blood. Although dexamethasone is commonly used in cases of severe bacterial infections (e.g., meningitis, community‐acquired pneumonia), controversy exists in its effectiveness in reducing mortality rates during *S. pneumoniae* infection (Silverman et al. 2004; O'Kane et al. 2006; Rodgers and Klugman 2011; Schuster et al. 2012). This study will determine the role of CRH and dexamethasone in mediating mortality, and how they influence inflammatory responses during pneumococcal infection.

## Materials and Methods

### Animals

Adult (6–8 weeks of age) female CD‐1 mice (Harlan Sprague Dawley, Indianapolis, IN) were used in all studies. Mice were maintained under specific pathogen‐free conditions on a 12:12‐h light/dark cycle (7:00 pm–7:00 am). Mice were kept under optimal temperature and humidity controlled conditions. The University of North Texas Health Science Center's Institutional Animal Care and Use Committee (IACUC) approved these studies.

### Intranasal infection and administration of pharmacologic agents

*Streptococcus pneumoniae* strain #6301 (ATCC, Manassas, VA) was grown for 16 h to obtain mid‐log phase cultures on blood agar plates (Thermo Fisher Scientific, Lenexa, KS). Mice were infected with 1 × 10^5^ colony‐forming units (CFUs) (LD_50_) of *S. pneumoniae* strain #6301 (ATCC, Manassas, VA) by intranasal route in a volume of 40 μL of brain–heart infusion broth (EMD Chemicals, Inc., Gibbtown, NJ) or broth (e.g., sham infection) after anesthesia (100–150 μL ketamine/xylazine intraperitoneally).

Human corticotropin‐releasing hormone, antalarmin, and dexamethasone were from Sigma‐Aldrich, St. Louis, MO. Optimal doses of CRH (1 mg/kg), antalarmin (1 mg/kg), and dexamethasone (1 mg/kg) were administered by intranasal route based on previous published results (Kim et al. 2011).

### Brochoalveolar lavage fluid isolation

Brochoalveolar lavage fluid (BALF) was prepared by intratracheal perfusion of 1 mL of sterile 1× phosphate‐buffered saline (PBS) solution using a 25‐gauge (G) blunt‐end needle. After removing cells by centrifugation, the total number of leukocytes were collected from BALF. BALF cells were quantified by viable cell trypan blue staining and hemocytometer techniques using light microscopy under 40× magnification. The noncellular BALF samples were stored at −80°C until analysis.

### CXCL1 chemokine detection in the BALF

CXCL1 chemokine and IL‐17A production was determined by sandwich ELISA method from BALF. All procedures were performed as described by the manufacturer (R&D Systems, Inc., Minneapolis, MN). Briefly, flat‐bottomed 96‐well plates were coated with an optimal titration of capture antibody followed by overnight blocking using 10% FBS in PBS to deter nonspecific binding. After incubation of samples at 4°C for 16 h, plates were incubated with biotin‐conjugated detection antibody and streptavidin–HRP (horseradish peroxidase). Tetramethylbenzidine (TMB) peroxidase substrate solution (Rockland Immunochemicals, Inc., Gilbertsville, PA) was added to each well for colorimetric determination of concentration of each cytokine according to standard curve generated by reference concentration of cytokine at a wavelength of 450 nm detected by colorimetric plate reader (Bioteck Instruments, Inc., Winooski, VT). ELISA antibody sets and recombinant cytokines were purchased from R&D Systems, Inc., Minneapolis, MN.

### Neutrophil depletion

Twenty‐four hours prior to infection, all treatment groups were administered a single injection of 0.5 mg of 1A8 neutralizing monoclonal antibody (Bio X cell, West Lebanon, NH) in a 200‐μL volume intraperitoneally. The neutralizing antibody was prepared in 1× PBS immediately preceding administration to animals. Efficiency of neutrophil depletion was confirmed by flow cytometry using LY6G antibody labeling of lung leukocytes. The average percent depletion of LY6G^+^ lung leukocytes from mice administered the 1A8 neutralizing antibody was 97.3 ± 2.3% (*n* = 5 mice per group). LY6G^+^ staining of lung leukocytes isolated from control mice administered IgG isotype‐matched antibody was 3.8 ± 1.2% (*n* = 5 mice per group) as determined by analysis of variance (ANOVA).

### Statistical analysis

Statistical analysis was performed using GraphPad Prism version 5.0p (GraphPad Software, San Diego, CA) and Stata 14 (StataCorp LP, College Station, TX). Log‐rank (Mantel–Cox) test was used for survival analysis. For multiexperimental group analysis, data were subjected to one‐way ANOVA. For two‐group comparisons, two‐sample proportion test was used. All data are expressed as mean ± standard error of mean (SEM). The two‐tailed level of significance was set to a *P* ≤ 0.05 for group difference.

## Results

**Figure 1:**
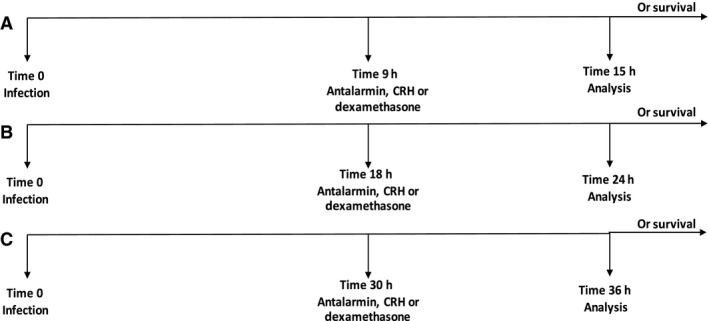
Experimental design. All mice were infected with 1 × 10^5^ colony‐forming units (CFUs) of *Streptococcus pneumoniae* by intranasal route. Selected groups of mice were administered PBS (placebo), antalarmin (1 mg/kg), CRH (1 mg/kg), or dexamethasone (1 mg/kg) by intranasal route at designated time points 9 h (A), 18 h (B), and 30 h (C). Selected groups of mice were sacrificed 6 h after administration of drug for analysis. Additional groups of mice were monitored for survival.

Reducing bacterial burden within a patient diagnosed with pneumococcal pneumonia is the main objective when determining treatment. However, during severe infection, the inability to clear infections due to deficient immune responses and ineffective antimicrobial treatment can lead to unwanted inflammatory reactions that require adjunctive anti‐inflammatory therapy. Under such circumstances, dexamethasone use has been shown to be less effective in reducing mortality risks among patients with infectious pneumonia (Ganceviciene et al. 2009; De Pascale et al. 2011; Meijvis et al. 2011). Our laboratory has previously demonstrated that intraperitoneal (i.p.) administration of antalarmin, a CRHR1‐specific antagonist reduces survival using a model of aversive stress plus *S. pneumoniae* infection (Kim et al. 2011). Although changes in disease outcome were seen when antagonists were administered i.p., no studies have investigated how targeting CRH and CRHR1 activity in the respiratory tract impacts disease outcome. Using an experimental model of murine respiratory *S. pneumoniae* infection, we compared sham‐treated mice to experimental groups of mice receiving intranasal administration of CRH, a CRHR1‐selective antagonist (antalarmin) or dexamethasone (Fig. 1). Dexamethasone is the pharmacologic glucocorticoid analog currently used clinically (Calogero et al. 1991; Kruisbeek 2001; Mogensen et al. 2008).

**Figure 2:**
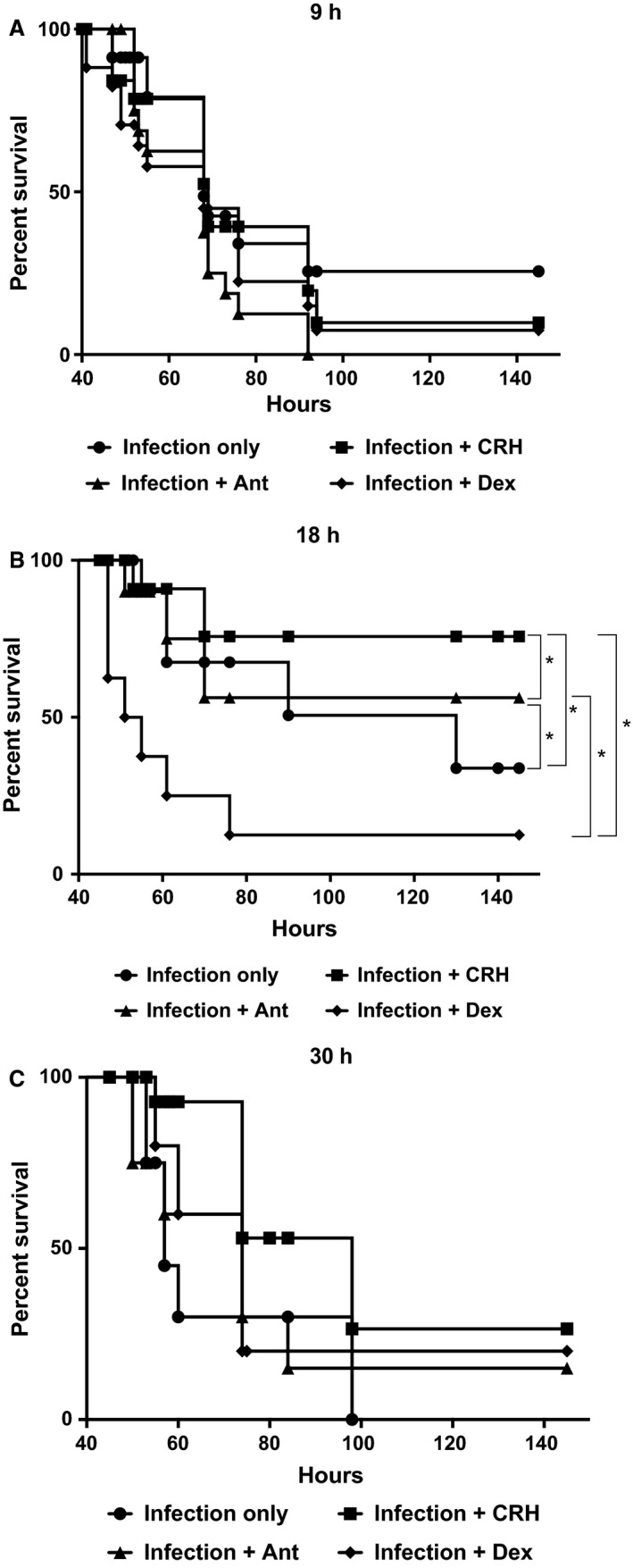
Time‐dependent administration of CRH effect on survival. Groups were administered antalarmin (1 mg/kg) CRH (1 mg/kg) or dexamethasone at a concentration of 1 mg/kg. All groups were compared with the infection‐only group that received placebo treatment (A) 9 h after infection, (B) 18 h after infection, and (C) 30 h after infection. *n* = 10 mice/group; **P ≤ 0.05 ANOVA or no significance. Software: Graph Prism.

Initial studies investigated the time‐associated effect of CRH administration during pneumococcal infection. Figure 2 shows the results of a survival study where groups were administered treatment at 9 (A), 18 (B), or 30 (C) h following infection. As shown in Figure 2A, survival was greatest among untreated mice. However, no significant differences in mortality rates were observed between experimental groups compared to sham‐treated mice. In contrast, mice administered CRH 18 h after infection resulted in a significantly higher survivorship compared to all experimental groups. Interestingly, intranasal administration of antalarmin significantly reduced the protective effect of CRH, suggesting that endogenous ligation of CRH to its cognate receptor CRHR1 could be mediating responses during pneumococcal infection (Fig. 2B). No significant differences in outcome were found between experimental groups at 30 h (Fig. 2C).

**Figure 3:**
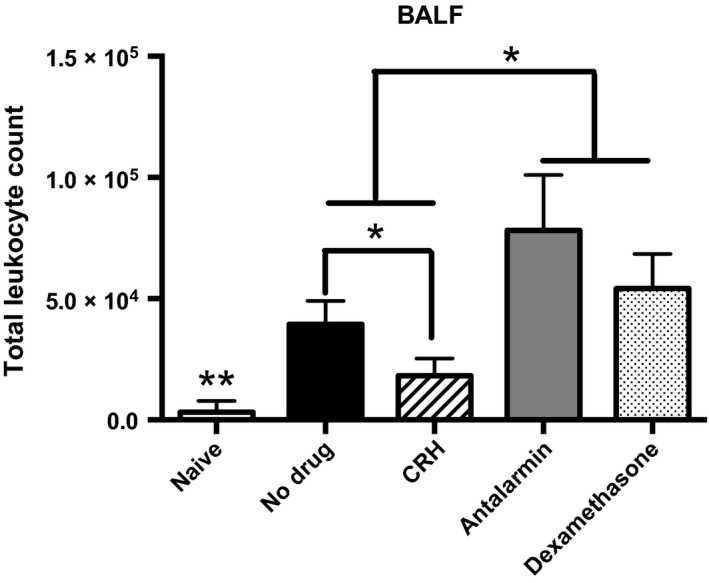
Corticotropin‐releasing hormone decreases total leukocyte numbers in the bronchoalveolar lavage fluid (BALF). CRH (1 mg/kg), antalarmin (1 mg/kg), or dexamethasone (1 mg/kg) was administered by intranasal route 18 h after infection. Total leukocytes numbers were determined in BALF of naïve and experimental groups of mice (*n* = 5). **Significant differences from all experimental groups, *P* ≤ 0.05. *Significant differences between experimental groups determined by ANOVA,* P* ≤ 0.05. Software: Graph Prism.

We next tested the hypothesis that survival is predicted by the type of cellular inflammatory response produced during infection given intranasal administration of CRH. We determined the leukocyte response to respiratory *S. pneumoniae* infection by enumerating the total leukocyte numbers in the BALF given intranasal administration of CRH, antalarmin, or dexamethasone. Mice administered CRH demonstrated a significant decrease in total leukocyte numbers compared to sham‐treated mice as well as mice administered dexamethasone. Accordingly, administration of antalarmin led to a significantly higher increase in the total leukocyte count compared to CRH and infection alone. Thus, affirming endogenous CRH ligation to CRHR1 as a mechanism of action (Fig. 3).

**Figure 4:**
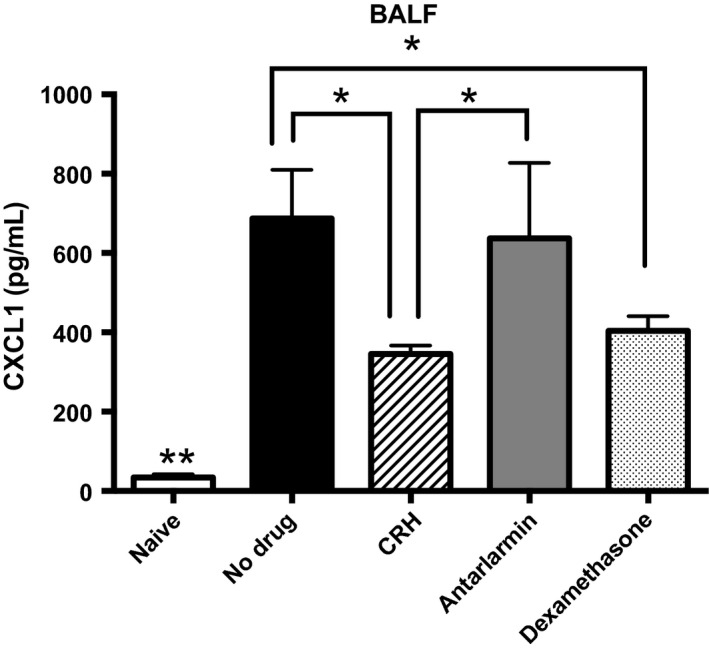
Production of CXCL1 in the bronchoalveolar lavage fluid (BALF) decreases with CRH. Naïve or following treatment, BALF was harvested from mice for chemokine evaluation. Using ELISA, CXCL1 production was evaluated. *n* = 5, statistical significance indicated by *. **Significant differences from all experimental groups, *P* ≤ 0.05. *Significant differences between experimental groups determined by ANOVA,* P* ≤ 0.05. Software: Graph Prism.

The above results suggest that CRH through triggering of CRHR1 could be a mechanism controlling cellular recruitment. CXCL1 is a chemokine released after the activation of epithelial cells and has been shown to preferentially lead to the recruitment of monocyte populations during pulmonary infection. We therefore determined how CRH and blocking its receptor would impact CXCL1 production along the respiratory airways. We found significantly lower levels of CXCL1 in the BALF among mice given CRH. Importantly, administration of the CRHR1‐selective antagonist antalarmin significantly increased CXCL1 levels below that found in the BALF of infected untreated mice and mice receiving dexamethasone (Fig. 4). These results demonstrate a relationship between CXCL1‐mediated neutrophil/monocyte recruitment and a CRH/CRHR1 mode of action. IL‐17A is also an important mediator of monocyte (e.g., macrophages, and neutrophils) recruitment along the respiratory tract (Zizzo and Cohen 2013; Bi et al. 2014). However, IL‐17A was not detected in the BALF among all experimental groups (data not shown).

**Figure 5:**
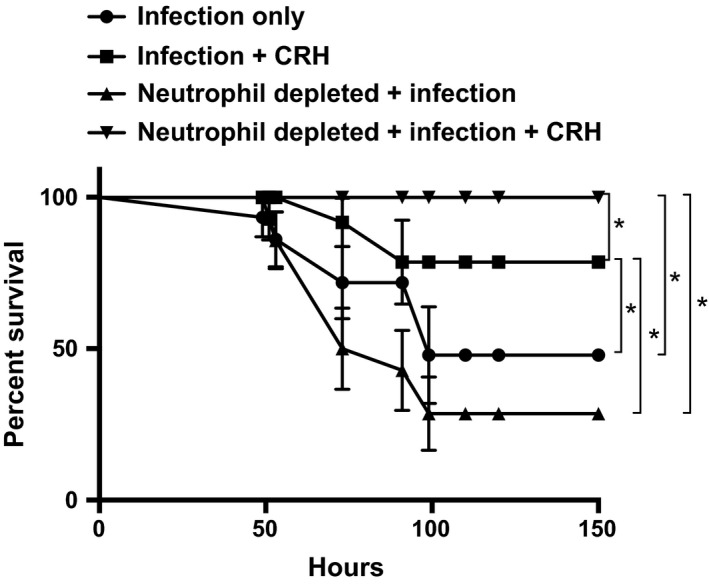
Elimination of neutrophils enhances survival with CRH. Mice were administered placebo or 1A8 neutralizing antibody (0.5 mg) by intraperitoneal route 1 day prior to *Streptococcus pneumoniae* infection (1 × 10^5^ CFU). Selected groups received CRH (1 mg/kg) by intranasal route 18 h after infection. All groups were compared with the infection‐only group that received placebo treatment. Data represent *n* = 5 or 10 mice/group. *Significant differences between experimental groups determined by Kaplan–Meier, *P* ≤ 0.05. Software: Graph Prism.

Neutrophils and monocyte/macrophage cells play a major role in mediating respiratory bacterial infections including *S. pneumoniae* (Whale and Griebel 2009; Costantini et al. 2011). Most intriguing of these immune cell types are their complex functional roles including the induction of potent proinflammatory and oxidative stress responses as well as their capacity to mediate anti‐inflammatory responses involved in disease resolution (Vakharia and Hinson 2005; Zhao et al. 2009). We have previously demonstrated a role for CRH in modulating leukocyte responses in lungs given restraint stress and *S. pneumoniae* infection (Kim et al. 2011). Specifically, those findings demonstrated divergent effects of CRH receptor antagonism on the influence of neutrophil and monocyte/macrophage infiltration to the lungs in response to infection. Due to their significant involvement in mediation pneumococcal infection, neutrophil depletion studies were performed to ascertain the potential relationship between CRH and neutrophil‐mediated responses associated with disease outcome. Figure 5 demonstrated that survival could be significantly increased by CRH administration in immunocompetent mice. Interestingly, CRH administration in the absence of neutrophils resulted in the highest survival rate compared to all experimental conditions. This suggests that CRH's mode of action on neutrophil function may in part be a determinant of mortality risks (Fig. 5).

## Discussion

Corticotropin‐releasing hormone is produced by the HPA. Its ability to influence immune function is commonly associated with the release of adrenal corticotropic hormone (ACTH) from the adrenal glands, resulting in cortisol release that in turn translates into nonspecific immune suppression (Rodgers and Klugman 2011). Alternatively, CRH is also secreted in peripheral tissues (e.g., synovial tissue, gastrointestinal tract, placenta), where it is believed to modulate cellular immune and inflammatory responses through preferences in CRH receptors 1 and 2 activity (Peracoli et al. 2011; Zhu et al. 2011; Liu et al. 2014). To date, few studies have defined the role of CRH in the regulation of pulmonary cellular immune and inflammatory responses (O'Kane et al. 2006). A previous study also demonstrated that preferences in CRH receptor expression could be associated with asthma severity (Drescher and Bai 2013). We have previously determined that i.p. injection of CRH receptor antagonists can modulate severity of pneumococcal pneumonia in the presence of aversive stress. The results from this study demonstrate for the first time the protective effect of intranasal CRH administration through modulation of cellular inflammatory responses across the respiratory tract.

*Streptococcus pneumoniae* is the leading causative agent of community‐acquired pneumonia worldwide and is responsible for the highest mortality rates among the elderly, young, and immunocompromised (De Pascale et al. 2011). In addition to antibiotic resistance, aberrant immune and inflammatory responses are believed to be a key determinant of disease outcome (Culpitt et al. 1999). Specifically, studies have documented uncontrolled inflammatory reactions produced by neutrophils and monocytes to be active participants in exacerbated responses in an effort to eradicate *S. pneumoniae* infection (Pichichero and Almudevar 2016). We hypothesized that targeting CRH‐mediated effects to the respiratory tract will predict disease outcome through modulation of immune and inflammatory responses. Initial studies determined the timing of intranasal CRH administration following *S. pneumoniae* infection to effect survival. Figure 2A–C demonstrate the effect of intranasal administration of CRH on survival when given at 9, 18, and 30 h after *S. pneumoniae* infection, respectively. Figure 2A shows that CRH as well as dexamethasone administered at 9 h has no significant effect on survival compared to untreated mice. This outcome suggests that host responses presumably innate cellular mechanisms of immune defense are unresponsive to CRH and dexamethasone administered at this time point of *S. pneumoniae* infection. Our finding that administration of the CRHR1‐selective antagonist antalarmin also did not impact outcome suggests that endogenous CRH is at most having a negligible role during the early stages of infection, supportive of CRH's inability to impact survival given 9 h after infection. In contrast, Figure 2B demonstrates a significant protective effect of CRH administered at 18 h after infection compared to dexamethasone which demonstrated the lowest survival outcome. Furthermore, in that no significant differences in survival were demonstrated between all experimental groups when CRH, dexamethasone, or antalarmin was administered at 30 h further illustrates the significance in defining the specificity of temporal host responses against *S. pneumoniae* infection as a determinant of CRH's protective efficacy. Thus, the influence of CRH could be predicted by the type and intensity of inflammatory mediators involved during specific times of an ensuing infection. We have previously shown that blocking CRH–CRHR1 activity by i.p. injection improves survival in mice subjected to *S. pneumoniae* infection under aversive restraint stress (Kim et al. 2011). This finding contrasts with our current findings suggesting that route of administration (e.g., nasal vs. i.p.) can define CRH's mode of action. One potential explanation could be the distinct differences known between mucosal immune environment and that of systemic immune responses. Future studies are required for in‐depth determination of preferential CRH receptor expression along respiratory and nonmucosal tissues.

Previous studies have demonstrated the importance of identifying the optimal window of therapeutic efficacy pertaining to pharmacologic use in the management of aberrant inflammatory responses (Calogero et al. 1991; Meijvis et al. 2011; Skrupky et al. 2011). A robust cellular immune response is absolutely required in early host protection against invading *S. pneumoniae* infection (Huang et al. 2016; Pichichero and Almudevar 2016; Vissers et al. 2016). Therefore, studies were performed to correlate cellular immune responses against *S. pneumoniae* with survival given intranasal administration of CRH. In that, intranasal administration of CRH significantly reduced the number of total leukocytes found in the BALF compared to untreated mice demonstrates CRH's ability to modulate cellular inflammatory responses (Fig. 3). Conversely, intranasal administration of the CRHR1 antagonist antalarmin significantly increased leukocytes numbers. Thus, reinforcing CRH's role as a modulator of respiratory cellular responses based on CRHR1 specificity of action. Interestingly, intranasal administration of dexamethasone did not reduce total BALF numbers. If fact, BALF leukocyte numbers in dexamethasone‐treated mice were higher than untreated mice. Chemotactic factors play a major role in cellular recruitment and therefore are critical determinants of inflammatory responses. One possible mechanism through which CRH may mediate cellular immune responses could be the regulation of chemokine function. To further substantiate the role of CRH as a mediator of immune and inflammatory responses along respiratory tissues, we compared the level of CXCL‐1 and IL‐17A in the BALF between naïve (control) and experimental groups (infection and/or treated animals). CXCL‐1 and IL‐17A are preferential mediators involved in the recruitment of neutrophils and monocyte subpopulations to sites of infection (Bigorgne et al. 2016). Although IL‐17A was not detected in BALF, Figure 4 shows that infected mice administered CRH at 18 h results in a significant decrease in CXCL1. This finding correlated with CRH‐induced decrease in total BALF leukocytes, demonstrating a mechanistic link between CRH and cellular immune and inflammatory responses associated with respiratory *S. pneumoniae* infection. Such findings are impactful in light of the importance in managing inflammatory reactions during severe pneumococcal disease, particularly for at‐risk populations. These findings suggest that CRH was more effective in inducing an anti‐inflammatory phenotype than dexamethasone, a known suppressor of inflammatory responses. While inhaled glucocorticoids have proven therapeutic in management of respiratory inflammatory disease (McKeever et al. 2013; Poulos et al. 2013; Finney et al. 2014), CRH may also be an effective alternative. Future studies that determine the influences of CRH on the broader array of cytokine and chemokine mediators will provide further understanding of how CRH and its cognate receptors could be useful in manipulating pro‐ versus anti‐inflammatory responses.

There is emerging debate of whether neutrophils are absolutely necessary to purge the system of foreign invaders during early pneumococcal infection (Peracoli et al. 2011; Sutherland et al. 2014), or if neutrophils have a greater potential to promote detrimental effects during early pneumococcal infection (Balamayooran et al. 2010; Costantini et al. 2011; Isailovic et al. 2015). The generation of the 1A8 neutrophil‐neutralizing antibody (Verbanac et al. 1993) affords the ability to distinguish the roles of neutrophils against bacterial infection. Here, we took advantage of the 1A8 antibody to determine the contribution of neutrophils in survival against pneumococcal infection and whether its influence could be linked to CRH's protective role. In that, depletion of neutrophils did not result in a significant difference in overall survival compared to neutrophil‐competent mice raises an important question relating their contribution to protection against pneumococcal infection. In support, previous studies have raised a similar argument related to their role against *S. pneumoniae* and other respiratory infectious disease. For example, Cooper et al. suggest that the requirement for neutrophils in protection against pneumococcal infection may depend on disease severity (Herrod 1984; Cooper et al. 2013; Drescher and Bai 2013). Consistent with Figure 2, intranasal administration of CRH to neutrophil‐competent mice increased survival compared to untreated mice. Most intriguing, however, was the observation that CRH administration compensated for the absence of neutrophils, resulting in a significantly higher survivorship compared to neutrophil‐competent mice administered CRH. This finding suggests that neutrophils may be expendable in protection against *S. pneumoniae* infection. Alternatively, one might consider that the necessity of neutrophils during infection is tightly linked to disease status (e.g., bacterial burden, inflammatory condition). To date, the direct effect of CRH on neutrophils and other leukocyte populations remain largely unknown. Our preliminary studies of their influence in lung parenchymal tissue suggest that neutrophils and other monocyte lineages are responsive to CRH (our preliminary findings). Knowledge of how CRH modulates leukocyte subpopulations’ function will benefit our understanding of CRH as a mediator of anti‐inflammatory inflammatory responses.

In conclusion, our studies reveal the potential novel use of nasal delivery of CRH in control of overt inflammatory responses localized along respiratory tissues with the potential in reducing mortality risks associated with pneumococcal infection. Importantly, our results provide evidence of neutrophils’ dispensable role during pneumococcal infection, particularly when considering adjuvant therapy. To date, dexamethasone is a primary standard of care in adjuvant treatment of respiratory‐related and the management of systemic pneumococcal disease (Sutherland et al. 2014). However, its efficacy in reducing mortality, particularly for certain populations remains uncertain. For example, Remmelts et al. found that dexamethasone use among certain individuals with community‐acquired pneumonia produce very diverse cytokine responses with potential for adverse disease outcome (Remmelts et al. 2012; Sternberg et al. 1992). We believe that the development of novel approaches which tailor cellular immune and inflammatory responses is needed and a further understanding of the mechanisms through which CRH regulated immune and inflammatory responses may reveal improved adjuvant treatment that will eliminate mortality risks associated with pneumococcal infection.

## Compliance and Ethical Standards

This research was supported by an institutional intramural award #RI6095 from the Office of Sponsored Research, University of North Texas Health Science Center.

## Statement on the Welfare of Animals

All applicable international, national, and/or institutional guidelines for the care and use of animals were followed. All procedure performed in studies involving animals were in accordance with the ethical standards of the institution or practice at which the studies were conducted.

## Conflict of Interest

The authors declare that they have no conflict of interest.
